# Mechanical bowel displacement to enable safe microwave ablation of a peripheral hepatic tumor in Chilaiditi anatomy

**DOI:** 10.1016/j.radcr.2026.07.111

**Published:** 2026-09-03

**Authors:** Sami Liljedahl, Daniel Morcos, Charles Kitley

**Affiliations:** a School of Medicine, University of Missouri, 1 Hospital Dr., Columbia, MO 65212, USA; b Department of Radiology, University of Missouri, 1 Hospital Dr., Columbia, MO 65212, USA

**Keywords:** Hepatocellular carcinoma, Chilaiditi’s anatomy, Microwave ablation

## Abstract

Hepatocellular carcinoma is a primary liver cancer that is often treated with locoregional therapies such as radiofrequency ablation, cryoablation, or microwave ablation. These locoregional treatments can pose risk of thermal injury to surrounding structures such as the gallbladder, stomach, and bowel when located adjacent to a target lesion. Safe locoregional therapy can be further complicated when a patient has an anatomical variant such as Chilaiditi’s anatomy. We describe a case using a blunt-tipped trocar needle to mechanically displace interposed bowel loops adjacent to a peripheral hepatocellular carcinoma in a patient with a Chilaiditi anatomical variation, thereby facilitating safe and therapeutic ablation of the suspected lesion without injuring the interposed bowel.

## Introduction

Hepatocellular carcinoma (HCC) is a primary cancer of the liver that is often difficult to treat due to multiple factors such as location, tumor aggressiveness, and distribution throughout the liver, making it the second leading cause of cancer mortality in men worldwide [1]. HCC lesions on CT imaging are typically focal hypodense hepatic nodules that demonstrate hyperenhancement following injection of IV contrast on arterial phase imaging, with portal venous phase washout on delayed imaging [2,3]. Surgery is typically offered as first-line therapy for HCC, but many patients with HCC have co-morbidities associated with cirrhosis that limit their surgical candidacy. For select patients with percutaneously accessible HCC lesions, treatment options include microwave ablation (MWA), radiofrequency ablation (RFA), and cryoablation, with techniques such as chemoembolization and radioembolization typically reserved for patients with extensive tumor burden, larger tumors, or tumors that are inaccessible for percutaneous ablation [4]. Ablation techniques, however, can damage structures adjacent to the treatment zone, such as bowel, stomach, gallbladder, and diaphragm [5], which poses additional risk for patients with a peripherally located HCC lesion or with anatomical variants.

Chilaiditi’s anatomy is an anatomical variant in which a segment of the colon is interposed between the diaphragm and the liver [6]. With the bowel adjacent to the liver in this anatomy, a peripheral hepatic tumor becomes even more difficult to treat locally with traditional percutaneous ablation techniques. Typical displacement methods for difficult anatomy with adjacent structures within the ablation zone include hydrodissection, carbodissection (CO_2_ displacement), and balloon interposition to create separation between the ablation zone and adjacent vulnerable structures [[7], [8], [9]]. Mechanical displacement of the stomach with blunt needles during hepatic ablation procedures has also been previously described [10], but application to bowel displacement in a Chilaiditi anatomical variant patient remains limited.

## Case report

**Fig. 1:**
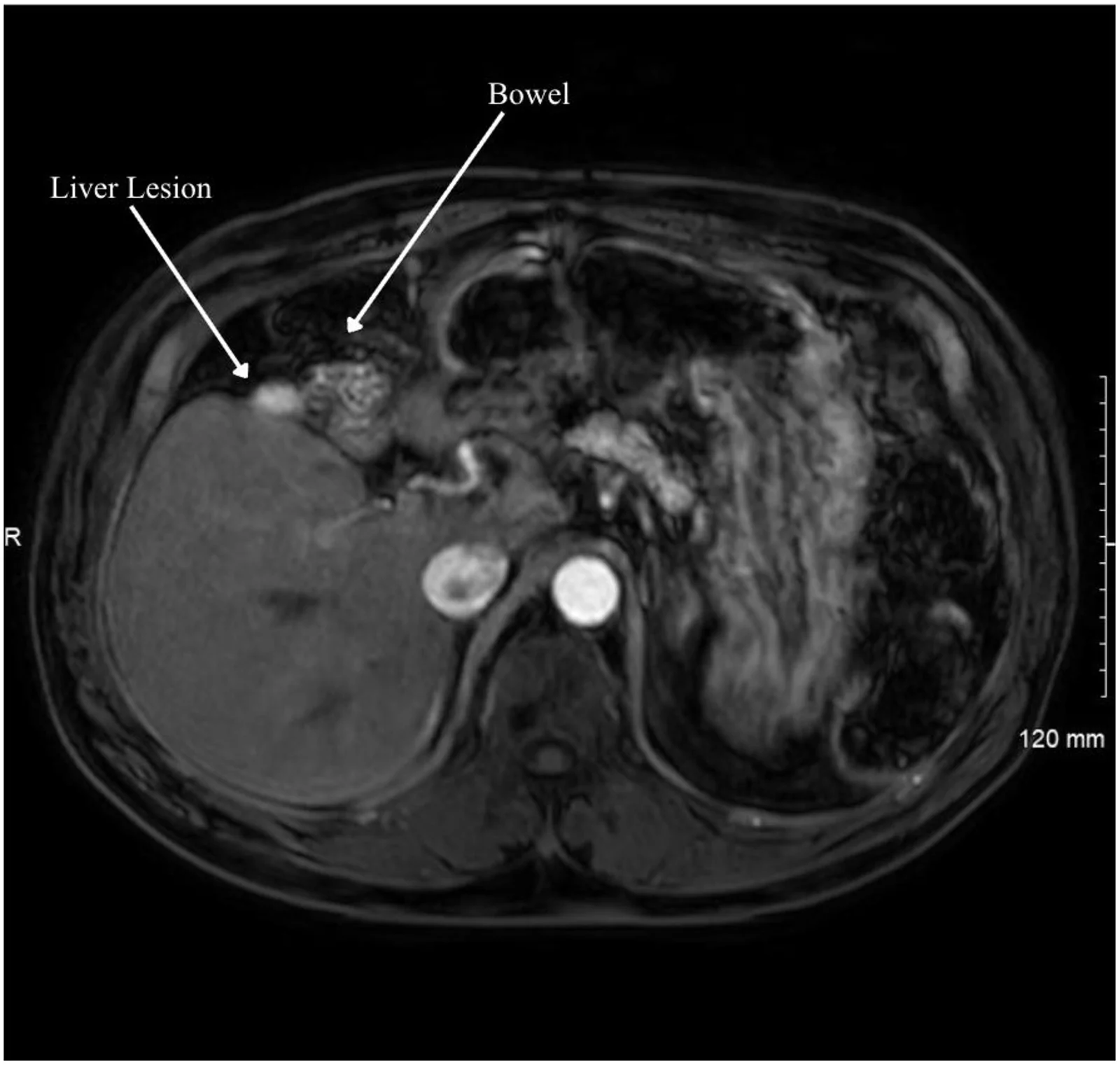
T1-weighted MRI abdomen demonstrating postcontrast arterial enhancement of a subcapsular exophytic hepatic lesion, with immediately adjacent large bowel.

A 75-year-old man with a past medical history of cirrhosis and a previously treated pancreatic neuroendocrine tumor was diagnosed with multifocal biopsy-proven HCC. Due to his clinical history of cirrhosis and locations of his HCC lesions, it was decided to pursue liver-directed treatment of his HCC. After biopsy of the lesions established an HCC diagnosis, his hepatic lesions were initially treated with Y90 radioembolization and subsequently percutaneous cryoablation of the recurrent lesion; however, on long-term follow-up, one partially exophytic lesion measuring 1.8 × 1.4 × 1.9 cm in segment IVa adjacent to a loop of interposed colon recurred on MRI imaging as evidenced by post-contrast arterial enhancement of the lesion (Fig. 1). This segment IVa recurrent lesion is consistent with LR-TR Viable disease due to nodular arterial-phase hyperenhancement within the prior ablation/treatment zone with corresponding washout. Based on lesion size and features and because the HCC is biopsy-proven and previously treated, a diagnostic LR-5 category is supplementary; the relevant characterization is the LR-TR Viable recurrence. This patient also had a Chilaiditi anatomical variant, evidenced on CT imaging with a loop of his large bowel interposed between his liver and diaphragm (Fig. 1). Due to his anatomy, this recurrent lesion had been previously treated with percutaneous CT-guided cryoablation and required hydrodissection to displace his adjacent large bowel.

Given the lesion location and prior treatment failure with both cryoablation and Y-90, microwave ablation was pursued as an alternative treatment modality. Previous left hepatic Y-90 radioembolization of the recurrent tumor in the IVa region of the liver had likely failed as the tumor rested in a watershed zone with arterial supply provided by both the right and left hepatic arteries. Given the lesions small size and prior failed embolization due to its dual arterial supply, other treatment modalities were discussed with the patient. The patient was given the option for surgical evaluation for resection but given his history of multiple prior treatments and known diagnosis of cirrhosis, he preferred pursuing further minimally invasive locoregional therapy if possible. MWA had not been previously performed on the lesion due to the immediately adjacent bowel and the risk of thermal injury; and given prior recurrence after attempts at cryoablation of the lesion it was felt to be the best alternative for attempts at percutaneous ablative treatment of the lesion.

It was decided that mechanical displacement of the adjacent bowel with a blunt trocar needle alone would be attempted to create a safe distance between the ablation zone since MWA can thermally damage structures, and this hepatic lesion was immediately adjacent to the large bowel loop. Mechanical displacement was preferred as an alternative to hydrodissection as previous attempts at displacing the loop of bowel using fluid during cryoablation provided suboptimal displacement. Given the location of the bowel within the peritoneum along the anterior surface of the liver, prior attempts at hydrodissection required significant volumes of fluid to maintain safe working space between the lesion and the loop of bowel, and the majority of the fluid tracked quickly away from the site into more dependent portions of the abdomen risking injury to the bowel loop.

**Fig. 2:**
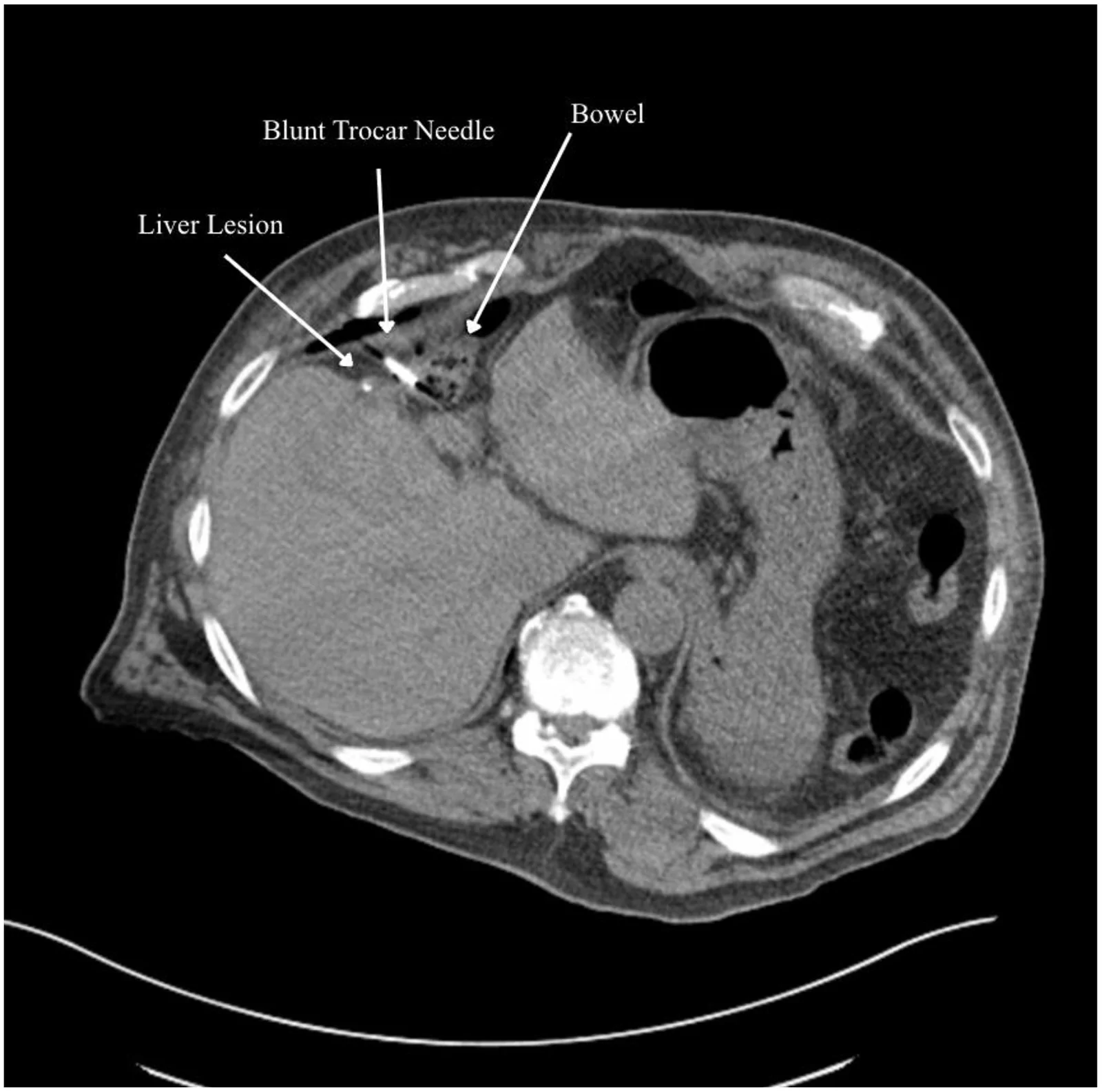
Subcapsular hepatic IVa segment lesion with closely associated colonic bowel loop (0 mm from the lesion) and blunt needle in place prior to mechanical displacement of the colon.

**Fig. 3:**
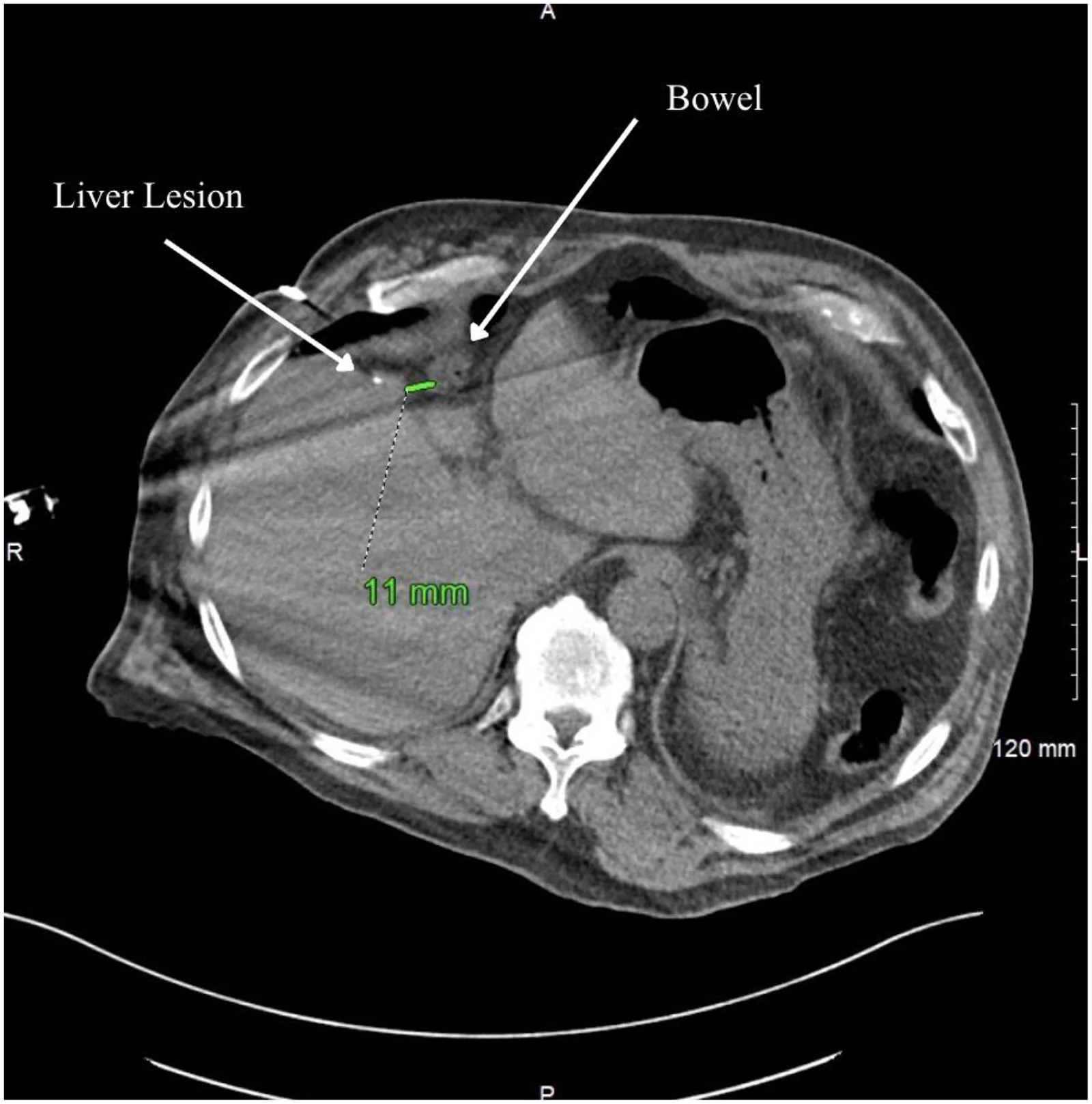
Interposed loop of colon displaced 11 mm away from the subcapsular hepatic IVa segment lesion following blunt needle displacement.

Following general anesthesia, procedural time-out, and sterile preparation, CT was used for guidance during the procedure, which confirmed colon interposed between the anterior liver surface and abdominal wall near the site of planned percutaneous access with a distance from the lesion of 0 mm (Fig. 2). The location of his hepatic lesion was also confirmed. A 17 g TruGuide (Bard, Murray Hill, NJ) trocar needle was then inserted under CT guidance into the adjacent peritoneal fat between the colonic serosa and liver capsule using sharp technique, and then the stylet was exchanged for the blunt stylet once the needle was positioned adjacent to the bowel and lesion. The blunt tip was then manipulated between the surface of the liver and the colon while a gentle sweeping motion was used to manipulate the bowel caudally and anteriorly under intermittent CT, thus displacing the interposed bowel loop away from the planned ablation zone by 11 mm (Fig. 3). The manipulation was performed with a leverage technique, using the needle entry site as a focal point for the sweeping motion. Displacement of the bowel was confirmed using CT before MWA was attempted.

**Fig. 4:**
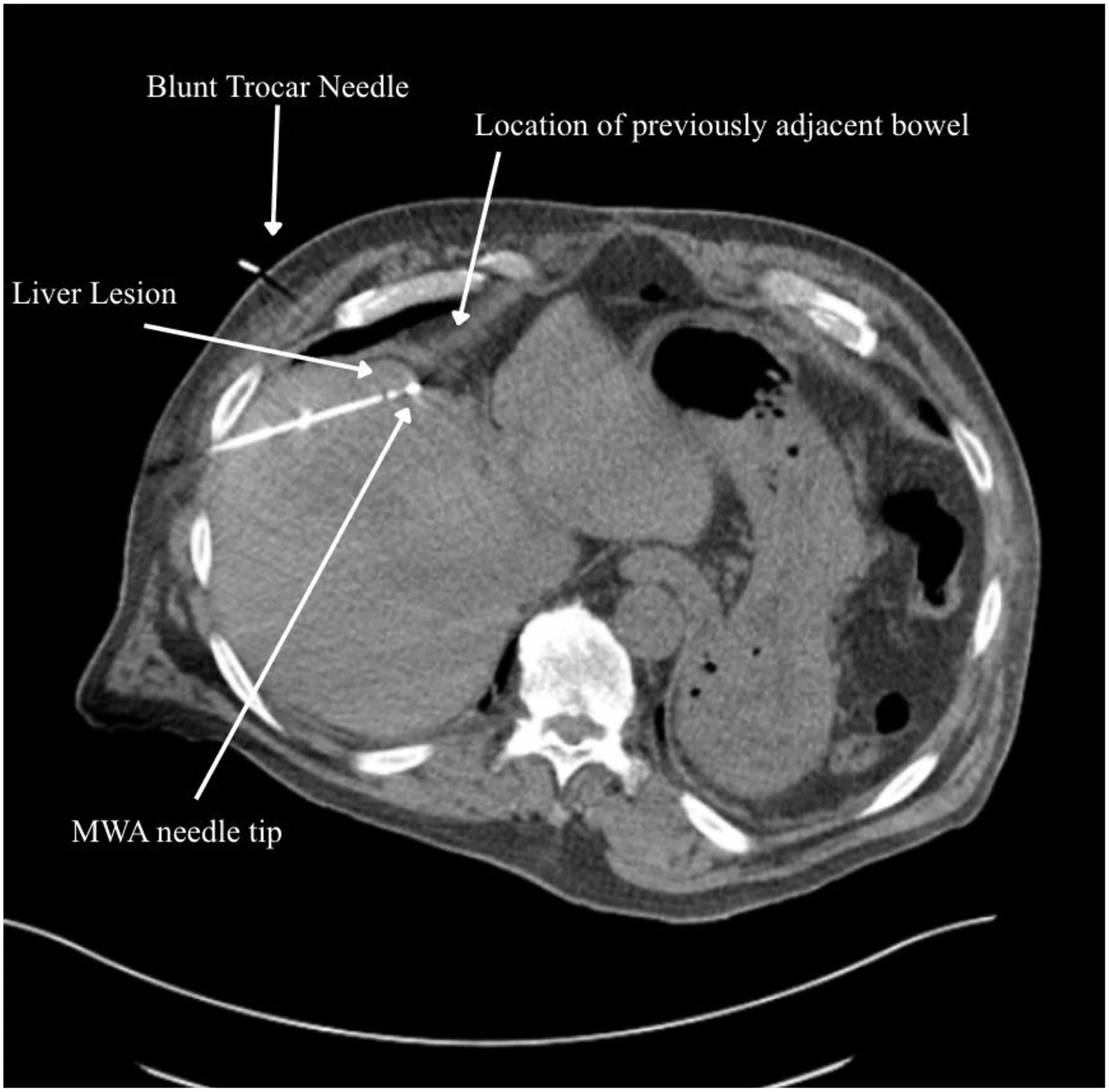
Ablation of the hepatic segment IVa lesion of interest. Note the previously adjacent bowel near the lesion has been displaced during ablation, as shown in Figure 3.

A 13-gauge, 15-cm Emprint (Medtronic Minneapolis, MN) MWA needle was then advanced into the target HCC lesion from a percutaneous approach using CT guidance (Fig. 4). The tip of the MWA ablation needle was placed with a posterior-lateral approach through the midportion of the lesion with the tip positioned along the anterior-medial aspect to ensure complete coverage in the expected ablation zone. At these settings, the ablation zone measures approximately 2.9 cm in maximal diameter, with 4 mm of ablation off the distal tip of the needle and 2.8 cm of ablation posteriorly from the tip of the needle. With the needle tip in the anterior-medial periphery of the hepatic lesion (Fig. 4), bowel was more than 7 mm from the absolute ablation zone, accounting for 4 mm of ablation off the end of the needle tip and 11 mm of distance of bowel from the lesion, so MWA ablation was performed at 75 watts for 4 minutes. This ablation had a predicted safety margin of at least 5 mm as the lesion measured 1.8 cm and the ablation zone was 2.9 cm in diameter. Postablation CT after the ablation demonstrated approximately 1 cm of antenna retraction, attributed to respiratory motion, which risked undertreatment of the deep margin. This was detected on immediate post-ablation CT by comparing antenna-tip position to the planned trajectory. The antenna was repositioned into the lesion, adequate bowel displacement was reconfirmed, and a second ablation cycle of 75 watts for 3 minutes was performed to cover the deficient margin. In all, this increased coverage of the ablation zone by using 2 adjacent/overlapping zones, which ultimately increased coverage of the tumor site. Final imaging confirmed complete coverage. With the liver lesion measuring 1.8 × 1.4 × 1.9 cm, the ablation diameter of 2.9 cm was deemed more than adequate for lesion treatment. During each ablation cycle, there was no separate intraprocedural thermometry as the Medtronic needle has a built-in safety shut off at 45 degrees Celsius.

**Fig. 5:**
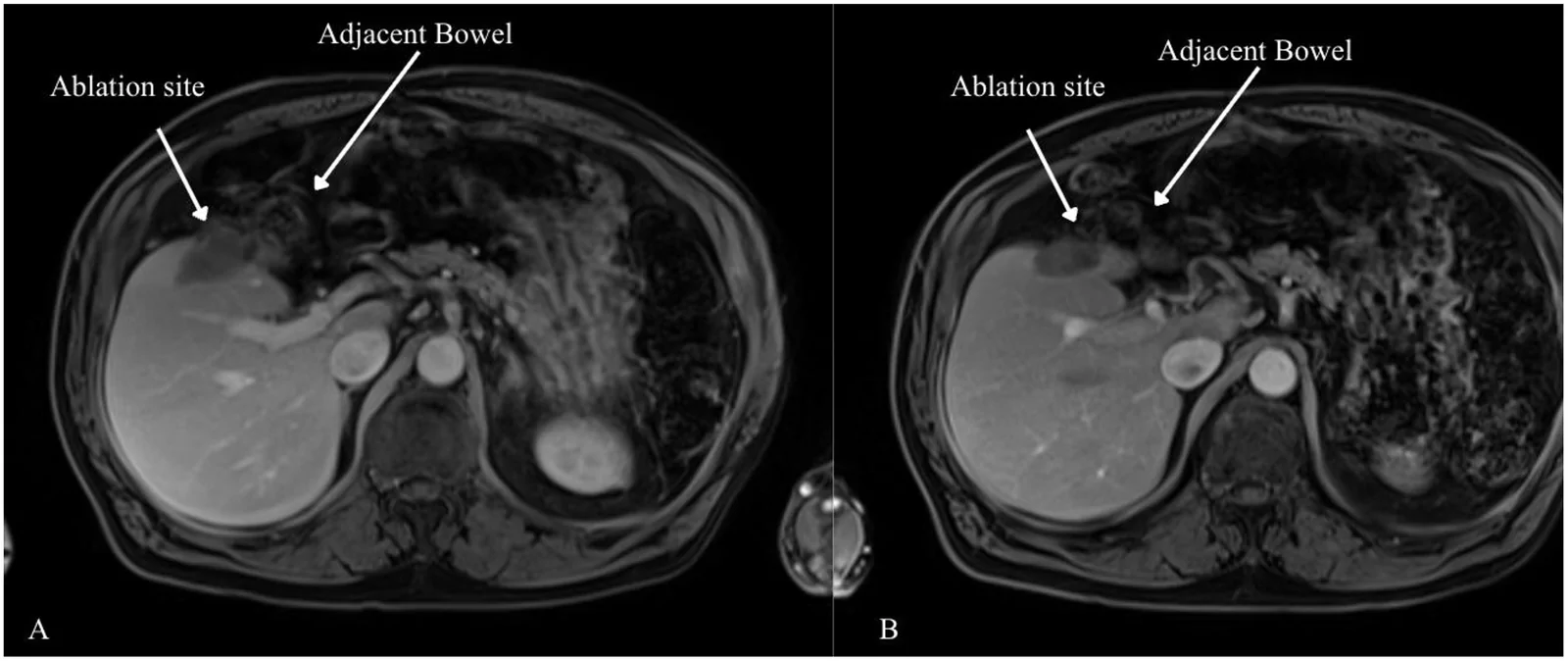
One-month (A) and 15 months (B) postprocedural MRIs demonstrating the segment IVa ablation cavity with no residual arterial enhancement and normal appearing adjacent large bowel.

Postablation imaging after the second cycle of ablation confirmed complete lesion coverage with maintained mechanical separation of the targeted lesion and ablation zone from the adjacent interposed bowel. It also demonstrated no injury to the bowel. The patient was monitored for 3 hours following the procedure and then was discharged home the same day without significant discomfort. During this observation period, his vitals remained stable, and he developed no peritoneal signs. On follow-up evaluation, the patient experienced no peri-procedural complications during his recovery and endorsed making a quick return to normal physical activities after the procedure. One-month follow-up MRI with IV contrast (Fig. 5A) showed no suspicious arterial enhancement or venous washout at the tumor site, with no evidence of adjacent bowel or diaphragmatic injury. Further follow-up imaging at 15 months has continued to show no suspicious arterial enhancement or venous washout at the tumor site, indicating the ablation of the hepatic lesion was successful (Fig. 5B).

## Discussion

HCC is a cancer of the liver that most often occurs in patients with comorbidities such as cirrhosis. This makes locoregional treatment such as MWA a favorable option in these patients [3]. Thermal ablation near the gastrointestinal tract or other adjacent critical structures requires advanced techniques to minimize injury to these non-target organs. These strategies typically include hydrodissection, carbodissection, or balloon interposition [[7], [8], [9]]. In Chilaiditi anatomical variation, colonic interposition increases the complexity of safe percutaneous access, especially in cases of exophytic liver masses adjacent to bowel which might lead to inadvertent thermal injury to non-target structures. Hydrodissection previously failed in this patient, which was presumed due to the intraperitoneal location of the tumor along the anterior liver surface, prompting the idea to attempt mechanical displacement using blunt needle technique as an alternative method for successful bowel displacement. Since mechanical displacement of the stomach had been previously utilized [10], this was felt to be a viable alternative to hydrodissection to maintain safe displacement while minimizing risk to the bowel from thermal energy in this non-dependent portion of the abdominal cavity.

Previous bowel displacement techniques described in the literature include the use of blunt-tip introducer sheaths or access needles as levers to physically reposition organs under real-time imaging. The technique of operator torquing to displace the stomach from the liver surface during ablation procedures to prevent inadvertent thermal injury has also been previously described [10]. This method may offer more predictable and immediate separation compared to fluid- or gas-based techniques, particularly when the bowel is tethered or when located within a confined intraperitoneal space, limiting fluid dispersion. Blunt trocar needle displacement also remains a safer displacement method for moving vital structures. In a study of 30 CT-guided lymph node biopsies that required use of a blunt trocar needle after a sharp needle was introduced into the peritoneum, there was no reported bowel injury and only one hematoma reported even when the trocar needle was used as a lever in a similar fashion to how it was used in this case [11]. This provides evidence of safety of using a blunt-ended trocar needle when moving bowel in a gentle fashion. The position of the needle in this case was also away from major vessels, decreasing risk of major vessel injury.

In the present case, gently sweeping the bowel caudally and anteriorly with a blunt-tipped trocar needle enabled targeted and stable displacement of the interposed colon, achieving a safe working window without the need of adjunctive fluid or gas dissection techniques. The bowel remained unharmed in this case, and the patient was able to be discharged the same day of the procedure. About 11 mm of distance from the lesion was also a comparable distance of separation to other displacement techniques, such as in a study of balloon displacement, in which they achieved 8-20 mm of stomach and bowel distance from hepatic lesions for microwave ablation [7]. The described case also had long-term treatment of the lesion, indicating the microwave ablation was successful, and the blunt trocar needle did not interfere with the intended treatment. This technique adds to the spectrum of available strategies and may serve as a viable option in cases where other modalities prove insufficient.

## Conclusion

Blunt needle displacement using CT guidance can enable safe microwave ablation of peripheral or exophytic hepatic lesions in patients with Chilaiditi’s anatomy and other anatomical variations. This technique may serve as a practical alternative when conventional displacement techniques are unsuccessful.

## Patient consent

Written, informed consent was obtained from the patient for publication prior to submission of this manuscript.
